# Glycine receptors in circulating white blood cells regulated by neuroinflammation

**DOI:** 10.3389/fimmu.2026.1749275

**Published:** 2026-02-23

**Authors:** Vikram Thakur, Yathip Mindy Chokpapone, Rakshak Mishra, Osarenoma Evbuomwan, Lizbeth Lopez, Camila Guerrero, Alexandra Chavez, Ingrid Carrizales, Brianna J. Rodriguez, Jonathan Lavezo, Gustavo J. Rodriguez, Huanyu Dou

**Affiliations:** 1 Department of Molecular and Translational Medicine, Paul L. Foster School of Medicine, Texas Tech University Health Sciences Center, El Paso, TX, United States; 2 Graduate School of Biomedical Sciences, Texas Tech University Health Sciences Center, El Paso, TX, United States; 3 Department of Pathology, Paul L. Foster School of Medicine, Texas Tech University Health Sciences Center, El Paso, TX, United States; 4 Department of Neurology, Paul L. Foster School of Medicine, Texas Tech University Health Sciences Center, El Paso, TX, United States

**Keywords:** blood biomarker, brain-GlyR-WBCs communication axis, neuroinflammation, non-neuronal GlyR, regulation of WBC GlyR expression

## Abstract

**Introduction:**

Neuroinflammation is involved in a wide range of neurological disorders, yet the lack of minimally invasive biomarkers hampers early diagnosis and therapeutic monitoring. Glycine receptors (GlyRs), classically known as inhibitory neurotransmitter receptors in the central nervous system, are increasingly recognized as regulators of immune signaling. Here, we identify GlyRs as novel peripheral indicators of neuroinflammation.

**Methods and results:**

We demonstrate that GlyRα1, α2, and α3 subunits are constitutively expressed in human and murine immune cells, with GlyRα2 predominating across peripheral tissues and the brain. Using ex vivo and *in vivo* mouse models, we found that the expression of GlyRα1, α2, and α3 in macrophages and circulating white blood cells (WBCs) was not directly mediated by inflammatory cytokine signaling in the brain or WBCs. Neuroinflammation upregulates GlyRα1 and α3 expression in the brain, spleen, bone marrow, and circulating WBCs. Immunostaining revealed GlyRα3 to the membrane and GlyRα1/2 to both the membrane and cytoplasm of WBCs. GlyR expression was also observed in the bone marrow, the spleen (macrophage-rich red pulp), and the neurons. Notably, GlyRα1 and α3 expression in WBCs was significantly elevated in neuroinflammation compared to control and systemic inflammation models. Changes in GlyR expression were not correlated with the expression of pro-inflammatory cytokines in the brain and WBCs. LPS-induced microglial (Iba1^+^) activation paralleled the upregulation of WBC GlyR, suggesting a reciprocal modulation between central and peripheral compartments.

**Conclusion:**

Together, these findings define a brain-glycinergic signaling-blood axis that maintains homeostatic protectivity. GlyR subunits, particularly GlyRα1 and α3, represent a neuropathology-induced modulation of GlyR signaling in peripheral immune cells.

## Introduction

Inflammation is a primary biological immune response to infection, cellular stress, or injury, involving a complex interaction among immune cells, cytokines, and signaling pathways. Although vital for host defense and tissue repair, dysregulated inflammation can lead to various pathological conditions, including cancer, autoimmune disorders, and neurodegenerative diseases. The inflammation of the central nervous system (CNS), known as neuroinflammation, is a hallmark of many neurological disorders, including stroke, Alzheimer’s disease, multiple sclerosis, Parkinson’s disease, and traumatic brain injury (TBI) (1). Neuroinflammation involves the activation of resident macrophages (microglia) and astrocytes, infiltration of immune cells, and the release of inflammatory mediators that disrupt neural functions. The systemic pathology of neuroinflammation remains poorly defined.

Glycine receptors (GlyRs) are pentameric proteins belonging to the Cys-loop family of ligand-gated ion channels composed of α and β subunits, primarily expressed in the brainstem and spinal cord, and mediate fast inhibitory neurotransmission by facilitating chloride ion influx, thereby regulating neuronal excitability (2, 3). Classically, glycinergic signaling is crucial in motor control, respiratory rhythm, sensory processing, and muscle tone both in health and disease. GlyRs play a complex, dual role in inflammation (4, 5). In neurons, GlyRs primarily mediate inhibitory neurotransmission. However, glycinergic signaling triggers immunomodulation in immune cells (6). Blocking GlyRs reduces neuroinflammation, opening new pathways for crosstalk between the brain and immune system (7).

GlyRs have recently attracted attention for their broader roles in transmitting pain signals (8) and in modulating neuro-immune interactions and inflammatory signaling within the CNS (9, 10). Given their clinical relevance, GlyRs are considered critical targets for managing chronic pain and inflammation by modulating glycinergic neurotransmission. The GlyRα3 subunit has emerged as a key player in the inflammatory pain pathways of the spinal cord dorsal horn (11). A higher density of the GlyRα3 subtype in the spinal dorsal horn correlates with the sensation of central inflammatory pain. Evidence also suggests that the GlyRα1 subtype within the spinal dorsal horn can be targeted to manage pain by inhibiting GlyR potentiation (12). Disruption of the inhibitory control of the glycinergic system leads to chronic inflammatory pain, highlighting the need for further research into the roles of β and α2 subunits in disinhibiting this system (13). Chronic inflammatory pain is triggered by glycinergic disinhibition of the spinal cord, involving synaptic α3β and GlyRα1β phosphorylation. Interneurons expressing PKCγ play a crucial role in pain circuits. About 80% of the inhibitory synapses of these interneurons contain GlyRα1 along with a γ-aminobutyric acid (GABA) receptor subtype (14), which could be explored as a potential pharmacological target. GlyRs may influence inflammatory responses by affecting cell activation, cytokine production, and intracellular ion homeostasis (15–17). Inflammatory cytokines, such as IL-1β, impact GlyR activity by inhibiting chloride currents (18), thereby modulating pain perception. Neurological conditions, such as stroke-induced injury, cause a decrease in GlyRα2; however, vascular endothelial growth factor/signal transducer and activator of transcription 3 (VEGF/pSTAT3) signaling can reverse GlyRα2 inhibition via glycine and protect the neurovascular supply (19). There are differences between GlyRs in adults and embryos. In the developing brain, GlyRs are present throughout, but in adulthood, they are limited to certain areas, including the retina and spinal cord. During early development, GlyRs are expressed in migratory neurons and dorsal progenitors (20). Additionally, the expression of these receptors varies by sex, with male mice showing higher overall GlyRα3 expression than female mice (21). Most studies on the role of GlyRs in inflammation and pain have been conducted within the CNS, highlighting an opportunity to explore their functions beyond the CNS.

New evidence indicates that GlyRs are also present in non-neuronal cells, including immune cells such as macrophages, microglia, and T lymphocytes (15, 17). Retinal ganglion cells (RGCs) in the mammalian retina display the presence of both GlyRα1 and GABAα3 receptors at the synapse, and early GABA expression is crucial for GlyR recruitment (22). Therefore, their presence in immune cells and their ability to regulate immune signaling pathways suggest an intriguing, underexplored role in systemic and neuroinflammation. Given GlyRs’ dual presence in the CNS and peripheral tissues, GlyRs may act as a bridge between neuroinflammatory events and observable changes in the peripheral immune system. In this study, we studied how non-neuronal GlyRs were regulated by the neuronal systemic and neuronal inflammatory mouse models with lipopolysaccharide (LPS). The neuroinflammation model was developed by intracranial injection of LPS. The systemic inflammation produced by intraperitoneal injection of LPS, which activates toll-like receptor 4 (TLR4) to release pro-inflammatory cytokines into the circulation, leading to systemic inflammatory responses (23). We investigated GlyR expression in peripheral tissues and WBCs to explore crosstalk between neuropathogenesis and systemic immune responses, potentially identifying a circulating WBC-based indicator of neuroinflammation. By comparing neuronal and systemic inflammation-induced GlyR expression profiles both *in vivo* and *in vitro*, we identified greater changes in non-neuronal GlyR expression under neuroinflammatory conditions. Our findings could lay the groundwork for creating a new brain-immune communication axis through GlyRs.

## Materials and methods

### Animal model and experimental procedure

Both male (**Supplementary Figures 1A–D**) and female mice were initially used to develop systemic and neuronal inflammatory models. No significant sex-dependent differences in GlyR expression were observed. This study was conducted on adult C57BL/6J mice (female, 5–8 weeks old, weighing 20–25 grams) after approval from the Institutional Animal Care and Use Committee (IACUC, protocol no. 11026/23002). The mice were housed in plastic cages under a 12-hour light/dark cycle, at 22 ± 2 °C and 50 ± 10% humidity, with free access to a pellet diet and water. All experimental procedures and animal welfare protocols were carried out in accordance with the Ethical Regulations on the Care and Use of Laboratory Animals at Texas Tech University Health Sciences Center, El Paso, and were approved by the IACUC committee for animal experiments.

### Systemic inflammatory mouse model

Systemic inflammation in C57BL/6J mice (n=4–6 mice) was induced by intraperitoneal (i.p.) injection of lipopolysaccharide (*LPS, #L2755; Sigma-Aldrich*) at a dose of 5 mg/kg body weight, freshly prepared in sterile phosphate-buffered saline (PBS). 2 and 5 days post-LPS injection, animals were euthanized with an i.p. injection of a ketamine-xylazine cocktail (100/16 mg/kg) following the American Veterinary Medical Association (AVMA) guidelines. Adequate anesthesia was confirmed by the absence of a withdrawal reflex to firm hind paw pressure. Blood samples were collected from the heart (cardiac puncture) in sterile EDTA vacutainers (Greiner, USA) to avoid clotting. Post cessation of breathing and heartbeat, cervical dislocation was done to ensure death. The brain, spleen, and bone marrow were rapidly removed, snap-frozen in dry ice, collected in sterile PBS (BM) on ice, and stored at -80 °C until further processing.

### Neuroinflammation mouse model

To develop a neuroinflammatory mouse model, mice were anesthetized with an i.p. injection of 100 mg/kg ketamine and 16 mg/kg xylazine. The mice’s toe pinch reflex was used to ensure the appropriate anesthesia. Each mice were injected with a stereotactic intracortical injection of LPS (12µg/5µl) into the left hemisphere of the brain (n=4–6 mice) for 2 and 5 days. In both cohorts, the mice had not received any treatment for inflammation. Post-surgery, the mice were monitored for sickness behavior and recovery. At the beginning of the experiment (day 0), following the AVMA guidelines, healthy mice (n=4-6) were sacrificed as controls. At the endpoint, mice were euthanized with an i.p. injection of a ketamine-xylazine cocktail (100/16 mg/kg), and the toe pinch reflex was used to confirm the deep anesthesia. A 25-30g needle on a 1ml syringe was inserted into the heart for blood collection. After blood collection, mice were verified as having no heartbeat and no breathing, and cervical dislocation was then performed to confirm death. The brain, spleen, and bone marrow were collected to study GlyRs and cytokine response.

### WBCs isolation

To prevent blood from clotting, heparin or EDTA-coated tubes were used to collect blood. The blood samples were treated with 10 mL red blood cell (RBC) lysis buffer and incubated for 5 min at room temperature. The cells were centrifuged at 300xg for 5 min., followed by two washes with cold PBS at 500xg for 5 min at 4 °C. The supernatant was aspirated, and then the WBCs were collected as a cell suspension for qRT-PCR and immunostaining.

### Human blood WBCs

Human PBMCs and CD14+ monocytes were purchased directly from BioIVT (HUMANCD14-0107684, HUMANPBMC-0002132, Westbury, NY, USA). These PBMCs and CD14+ monocytes from healthy individuals were used to perform RNA extraction for gene expression and immunofluorescence for GlyRs localization studies.

### Bone marrow cell isolation and LPS stimulation

To isolate the bone marrow from CB57L/6 mice, the surrounding musculature was excised to access the mouse femur. The proximal end of the femur, as well as just beyond its distal end, was cut to ensure complete removal. The femur was then placed in cold PBS to reduce and prevent cell death. Within a BSL-2 hood, residual tissue on the femur was removed. Surgical scissors were used to cut the distal and proximal ends of the femur, allowing access to the bone marrow in the central part of the bone. A 20-gauge needle was used to inject cold PBS through the femur to dislodge the bone marrow onto a sterile petri dish. The bone marrow was filtered through a 40 μm nylon mesh filter into a 50 mL Falcon tube, with the syringe’s rubber stopper used to homogenize the marrow through the filter. The filtered bone marrow suspension was centrifuged at 500×g for 5 minutes, and the PBS was then removed. To the sterile bone marrow cell pellet, 200 µL of RBC lysis buffer was added and incubated for 5 minutes. After centrifugation, the RBC lysis buffer was discarded, and the cell pellet was collected.

Mouse bone marrow cells were cultured in a medium containing DMEM with glutamine, a mixture of penicillin and streptomycin antibiotics, 10% fetal bovine serum (FBS), and 10% macrophage-colony-stimulating factor (M-CSF) to differentiate into bone marrow macrophages (BMMs). These cells were grown in a 6-well plate, on coverslips with or without LPS treatment.

### Quantitative real-time PCR

Total RNA was extracted from blood, bone marrow cells, brain, and spleen tissues using the TRIzol (#15596018, Thermo Scientific, USA) method following the manufacturer’s instructions. The extracted RNA was quantified and checked for purity with a NanoDrop 2000 spectrophotometer (Thermo Scientific, USA). An equal amount, specifically 2 µg of RNA from each sample, was used for cDNA synthesis with the high-capacity cDNA reverse transcription kit (#4368814, Thermo Scientific, USA). Mice and human-specific primers targeting different GlyRs, including GlyRα-1, GlyRα-2, GlyRα-3, and housekeeping genes β-actin and beta2-microglobulin (B2M) (Sigma Aldrich, USA), were used (**Table 1**).

**Table 1:** Sequences of the primers used for gene expression analysis.

Gene	Forward primer (5’-3’)	Reverse primer (5’-3’)
*M1_GlyRα-1*	AGAAAGACCTGAGATACTGC	ATGTACATCTGGATCAGGTAG
*M2_GlyRα-2*	CAAAGGTCTCCTATGTGAAAG	TCTTCTTCCTTATTCTGCCTC
*M1_GlyRα-3*	CCTCCTTTTTGTGTTCTCAG	AGCTTCCGTCTTATTCTTTC
*M2_GlyRα-3*	AATAAGACGGAAGCTTTTGC	CATTTCATCAGGGCTCTTTG
*M3_GlyRα-3*	ACTTACTATGACCACACAGAG	CCGTCTTATTCTTTCTCTTTCG
*M_β-actin*	AGAAGCTGTGCTATGTTGCTCTA	TCAGGCAGCTCATAGCTCTTC
*M1_gapdh*	CTAATGACCACAGTCCATTC	GATGGGATGATGTTTTGGTG
*M2_gapdh*	GACTATAACCCTGGCTCTATG	GAATGGACTGTGGTCATTAG
*H2_GlyRα-1*	TGGACTATAGGGTCAACATC	GTCGTCAGGGTATTCATTATAG
*H2_GlyRα-2*	AGGTCTCCTATGTAAAAGCG	TCCTTATTCTGCCTCTTCTG
*H1_GlyRα-3*	CCTCCTTTTTGTGTTTTCAG	TACCTCATCATCCATATCTGAG
*H_B2M*	AAGGACTGGTCTTTCTATCTC	GATCCCACTTAACTATCTTGG
*H2_GAPDH*	CTTTTGCGTCGCCAG	TTGATGGCAACAATATCCAC

Gene expression was determined in a 10µl reaction volume using 5µl of SYBR Green Master mix (1X) (*#KR0389, Kapa Biosystems, South Africa*), 0.5µl each of forward and reverse primer (10 µM), 3µl nuclease-free water, and 1µl cDNA. Roche Light Cycler 96 (*Roche, USA*) was used to run the reaction with the following thermal profile: pre-incubation at 95°C for 3 sec., followed by 43 cycles of amplification (denaturation at 95°C for 10 sec., primer annealing at 60°C for 1 min., and extension at 72°C for 2 sec.) with default acquisition at 72°C. Finally, the gene expression was calculated using the 2^^-ΔΔCt^ as relative fold change.

### Immunofluorescence

The freshly isolated WBCs, BM, splenic cells, and macrophages were fixed in 4% paraformaldehyde (PFA) on a slide and left to dry at room temperature. The snap-frozen brain and spleen tissues were embedded in cryomolds (Tissue-Tek, USA) using optimal cutting temperature (OCT) compound (Tissue-Tek, USA) and stored at -80°C. For each brain and spleen tissue, 5µm-thick sections were cut using a cryostat (Leica CM3050S, USA) set at -20°C. The tissue sections on slides were fixed with a 1:1 ratio of acetone and ethanol (-20°C).

The slides were rinsed with PBS, followed by permeabilization using 0.1% Triton. After blocking with 5% normal goat serum (NGS) for 1 hour, the slides and coverslips were stained with primary antibodies targeting human and mouse GlyRα1 (1:500, #NB300-113, Novus Bio, Littleton, CO), GlyRα3 (1:100, #75-417, Neuromab, UC Davis/NIH), CD11b (1:100, #Sc-1186, Santa Cruz Biotechnology), CD-68 (1:100, #Sc-20060, Santa Cruz Biotechnology), and Iba1 (1:400, #MABN92, Millipore Sigma) (**Table 2**) overnight at 4°C. After incubation, the slides were washed three times with PBS for 5 minutes each, then incubated with appropriate secondary antibodies, i.e., Alexa Fluor 488 (1:200) and/or 594 (1:200) (Thermo-Fisher Scientific) for 1 hour at room temperature. A DAPI mounting agent (#AB104139, Santa Cruz Biotechnology) was used for nuclear staining and mounting. Finally, immunofluorescence images were captured using a fluorescent microscope.

**Table 2:** Antibodies used for immunostaining.

Antibody	Make/company	Dilution used
GlyRα-1/2 (anti-rabbit)	NovusBio, Littleton, CO, #NB300-113	1:500
GlyRα-3 (anti-mice)	Neuromab, UC Davis/NIH, #75-417	1:100
CD11b (anti-rat)	Santa Cruz Biotechnology, #Sc-1186	1:00
CD68 (anti-rat)	Santa Cruz Biotechnology, #Sc-20060	1:100
Iba1 (anti-mice)	Millipore Sigma, #MABN92	1:500
Alexa Fluor 488	Thermo-Fisher Scientific	1:200
Alexa flour 594	Thermo-Fisher Scientific	1:200

### Fluorescent microscopy

Changes in the expression and localization of GlyRα subunits in WBCs, BMMs, spleen, and brain tissues were observed by imaging with a Nikon Ti-Eclipse microscope (Nikon Instruments Inc., Melville, NY). The 60x magnification lens with oil immersion was set for higher resolving power in the microscope. The NS Elements AR program was utilized to image the slides. Images were analyzed in ImageJ, processed in Adobe Photoshop, and compiled in Illustrator for publication.

### Statistical analysis

GraphPad Prism (version 7.0, GraphPad Software Inc., USA) was used for data analysis. For comparisons between two groups, an unpaired two-tailed Student’s t-test was applied. Multiple group comparisons were performed using one-way or two-way analysis of variance (ANOVA), followed by Tukey’s *post hoc* analysis. The data were presented as mean ± S.E.M. Fold change in gene expression was calculated using the 2^^-ΔΔCt^ method with control samples normalized to 1. All the experiments were repeated 2–3 times in duplicates. Statistical significance was defined as p<0.05.

## Results

### Expression of GlyRs in human WBCs

**Figure 1:**
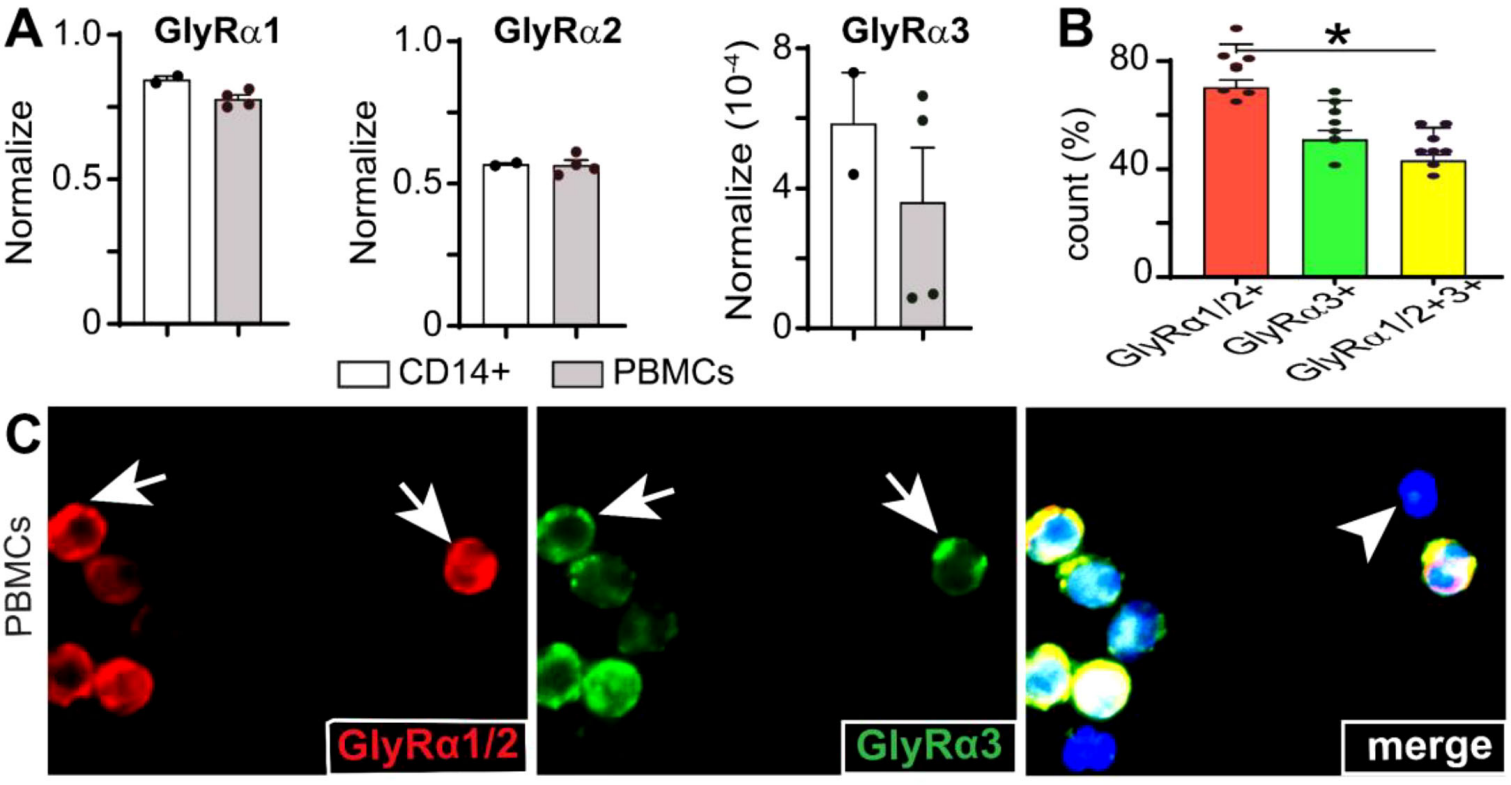
Identification and localization of GlyRα subunits in human PBMCs and CD14+ monocytes. **(A)** Relative mRNA expression of GlyRα1, α2, and α3 in CD14+ monocytes (white bars) and PBMCs (gray bars), normalized to β2-microglobulin housekeeping gene. GlyRα1 showed the highest expression, followed by GlyRα2 and α3. Data are presented as mean ± SEM **(B)** Quantification of PBMCs expressing 55.21 ± 2.8% of GlyRα1/2+ cells and 46.87 ± 3.4% of GlyRα3+ cells, with 43.12 ± 2.2% of GlyRα1/2+/GlyRα3+ PBMCs. GlyRα1/2+ cells were significantly more numerous than dual-positive cells (p=0.015*). Statistical significance was assessed using one-way ANOVA; bars depict mean ± SEM; *p<0.05. **(C)** Representative immunofluorescence images of PBMCs showing GlyRα1/2 (red), GlyRα3 (green), and nuclei stained with DAPI (blue, arrowhead). The merged panel shows the co-localization of GlyRα1/2 and GlyRα3, with a few cells negative for GlyRs.

Although GlyRs are extensively characterized and studied in the brain and spinal cord, their expression and functional significance in circulating immune cells remain largely unexplored. We investigated and provided evidence that human circulating white blood cells (WBCs), specifically peripheral blood mononuclear cells (PBMCs) and CD14+ monocytes, express transcripts and proteins corresponding to different GlyRα1, α2, and α3 subunits. A qRT-PCR assay was used to detect the gene expression of GlyR α1, α2, and α3 subunits in PBMCs and CD14+ monocytes collected from healthy individuals. Among these, GlyRα1 (0.77 ± 0.01 and 0.84 ± 0.01) and GlyRα2 (0.56 ± 0.01 and 0.56 ± 0.01) subunits showed the highest expression in PBMCs and CD14+ monocytes, respectively, whereas GlyRα3 was expressed at lower levels (**Figure 1A**).

Next, protein expression of GlyR was detected in human PBMCs, immunostaining with antibodies against GlyRα1/2 (*red*) and GlyRα3 (*green*). Nuclear DNA was stained with DAPI (*blue*). Quantitative images revealed 55.21 ± 2.8% of GlyRα1/2+ cells and 46.87 ± 3.4% of GlyRα3+ cells with 43.12 ± 2.2% of GlyRα1/2+/GlyRα3+ CD14+ monocytes (**Figure 1B**). Immunofluorescence results indicated that GlyRα1/2 (**Figure 1C**, red) and GlyRα3 (**Figure 1C**, green) are present both in the cytoplasm (16.45% and 7.59%) and on the membrane (73.41% and 49.36%) of PBMCs, respectively. Additionally, the merged image exhibited the co-expression of GlyRα1/2 and GlyRα3. The cellular distribution of GlyRα1/2 and GlyRα3 suggested possibilities of receptor assembly and localization, similar to neuronal GlyRs.

### Identification of GlyRs in the mouse CNS and peripheral immune system

**Figure 2:**
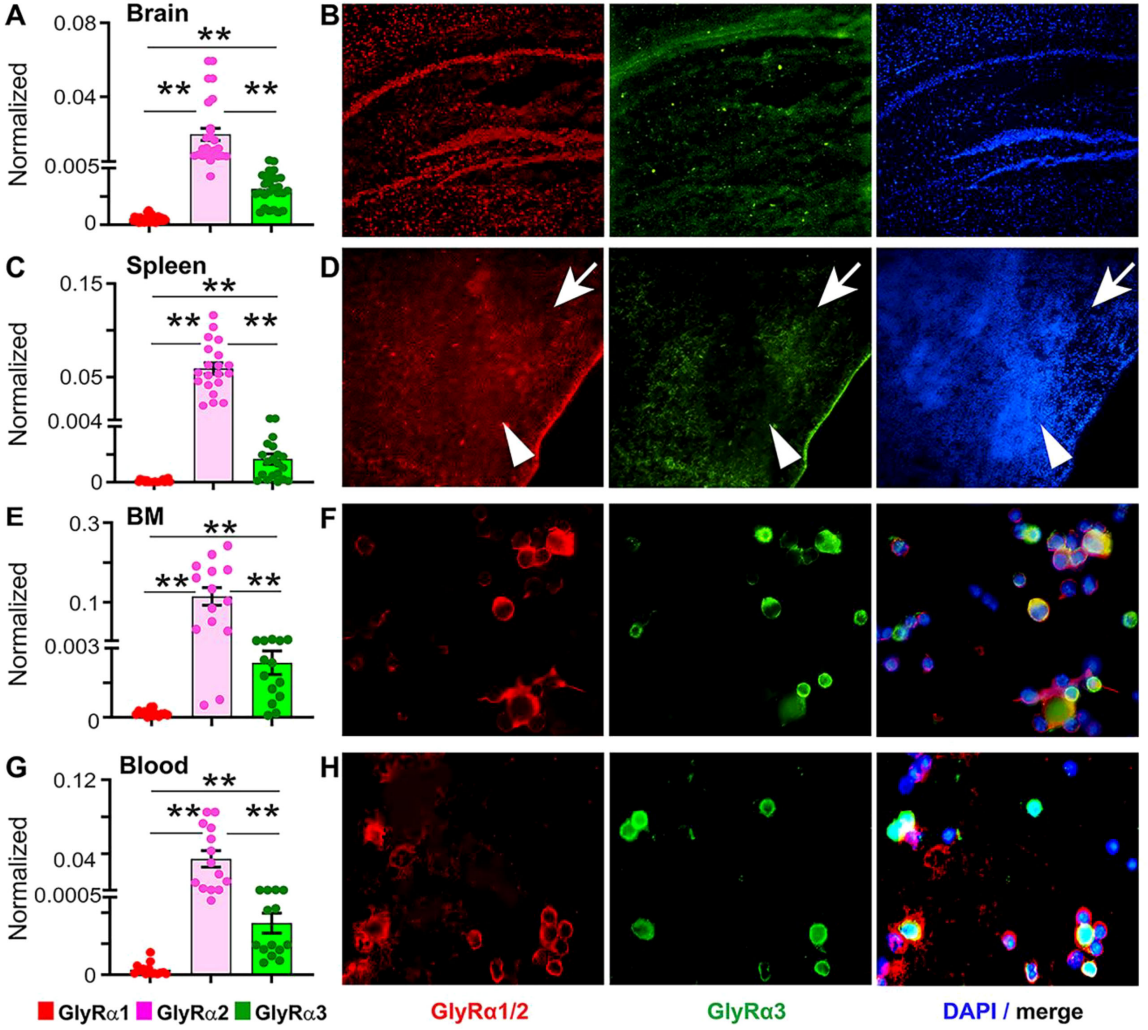
Identification of GlyRα subunits in mouse brain, spleen, bone marrow, and blood. Normalized mRNA expression levels of GlyRα1, α2, and α3 in mouse brain **(A)**, spleen **(C)**, bone marrow (BM; **E**), and blood **(G)** show the highest expression of GlyRα2, followed by GlyRα3 and α1. Statistical significance was determined using one-way ANOVA followed by Tukey’s *post hoc* test. Data are shown as mean ± SEM; p<0.01** (n=4-6 mice per group). **(B)** Representative immunofluorescence images of brain sections stained for GlyRα1/2 (*red*), GlyRα3 (*green*), and nuclei (DAPI, *blue*). GlyRα1/2 and α3 signals show co-expression within neurons of the hippocampus. **(D)** Spleen sections display strong GlyRα1/2 (red) staining in both red (arrow) and white pulps (arrow head), whereas GlyRα3 (green) staining is localized to the macrophage zone of red pulp. **(F, H)** Immunostaining of BM and WBCs shows strong membrane expression of GlyRα1/2 and α3. GlyRα3 staining marks distinct cell populations distributed in the cytoplasm and membrane. The merged image shows GlyRα1/2 and α3 co-localization, indicating heterogeneity in receptor subunit expression among different cell BM and WBC subsets.

After examining GlyR expression in human WBCs, we were interested in assessing the gene expression levels of GlyRα1, α2, and α3 in the mouse brain (**Figure 2A**) using qRT-PCR. GlyRα2 showed a statistically significant higher expression (0.019 ± 0.003, p<0.01**), followed by GlyRα3 (0.003 ± 0.0002, p<0.01**) and GlyRα1 (0.0005 ± 5.446e-005, p<0.01**). Immunostaining of brain sections showed co-expression of GlyRα1/2 (**Figure 2B**, red) and GlyRα3 (**Figure 2B**, green) mainly within neurons of the hippocampus (**Figure 2B**).

Next, we detected the expression of GlyRs in the peripheral organ spleen, where GlyRα2 remained dominant (0.05 ± 0.006, p<0.001**), followed by GlyRα3 (0.001 ± 0.0003) and GlyRα1 (8e-005 ± 2.069e-005) expression (**Figure 2C**). Immunostaining of spleen tissue showed strong GlyRα1/2 expression (**Figure 2D**, red) in both red (*arrow*) and white pulps (*arrow head*), whereas GlyRα3 localized to the macrophage zone of red pulp (**Figure 2D**, green, arrow).

Circulating WBCs and bone marrow cells (BM) were also analyzed for identifying GlyRα expression. BM cells and WBCs showed dominance of GlyRα2 expression, followed by GlyRα3 and GlyRα1 (**Figures 2E, G**). Immunostaining revealed that GlyRα1/2+ (*red*) and GlyRα3+ (*green*) were more dominantly expressed on the membrane, with less expression in the cytoplasm of BM and WBCs (**Figures 2F, H**). This is in accordance with the study of *Eynden et al.* (24). The results show that BM and WBCs express the GlyRα subunits, albeit at different baseline levels.

### Inflammation regulates GlyR expression in monocytes/macrophages

**Figure 3:**
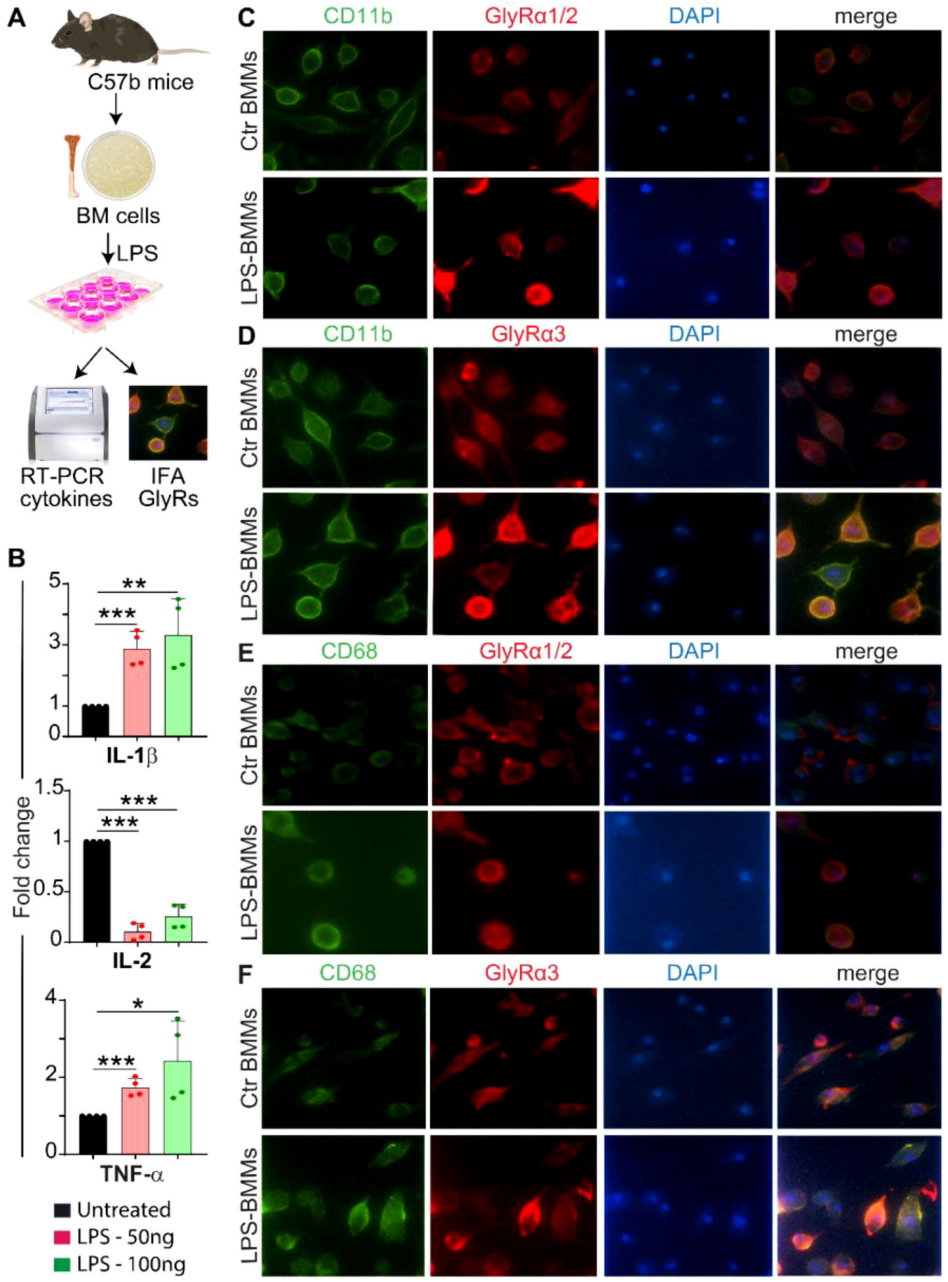
GlyRα subunit response to LPS-treated monocytes and neutrophils **(A)** Experimental workflow showing BM cells from C57BL/6 mice were differentiated into macrophages (BMMs), treated with LPS (50 and 100 ng/mL), and analyzed by qRT-PCR and immunofluorescence. **(B)** LPS-induced dose-dependent increases in IL-1β and TNFα expression with IL-2 downregulation, confirming macrophage activation. Data was calculated using the 2^^-ΔΔCt^ method (mean ± SEM) and analyzed using one-way ANOVA (*p<0.05, **p<0.01, and ***p<0.001). **(C, D)** Representative immunofluorescence images showing expression and co-localization of GlyRα1/2 and 3 (*red*) with CD11b+ monocytes/neutrophils (*green*). The enhanced GlyRα1/2 and α3 immunoreactivity in LPS-treated CD11b+ cells indicates activation-dependent upregulation. **(E, F)** Representative immunofluorescence images showing staining and colocalization of the macrophage marker CD68 (*green*) with GlyRα1/2 **(E)** and α3 (**F**, *red*) in control and LPS-treated BMMs. During inflammation, a noticeable increase in GlyRα1/2 and α3 fluorescence intensity was observed at the cell periphery, co-localizing with CD68, indicating possible increased receptor surface accumulation or transcriptional upregulation upon activation.

Inflammation-regulated dynamic changes in GlyR expression were analyzed using the LPS-induced inflammatory macrophages model. Firstly, monocytes/macrophages were treated with LPS, and then they were examined using an immunofluorescence assay (**Figure 3A**). Monocytes showed LPS-dose-dependent significant fold increase in IL-1β and TNFα with a decrease in IL-2 expression (**Figure 3B**). In CD11b+ monocytes and neutrophils, stronger co-expression with GlyRα1/2 (**Figure 3C**) and GlyRα3 (**Figure 3D**) was observed in LPS-treated monocytes compared to the control. LPS-activated monocytes exhibited greater expression of CD68 in parallel to an increase of GlyRα1/2 (**Figure 3E**) and GlyRα-3 (**Figure 3F**). The modification of GlyR expression at protein levels suggests that GlyRs may play a functional role in innate immunity.

### Brain GlyR is regulated by systemic and neuronal inflammation

**Figure 4:**
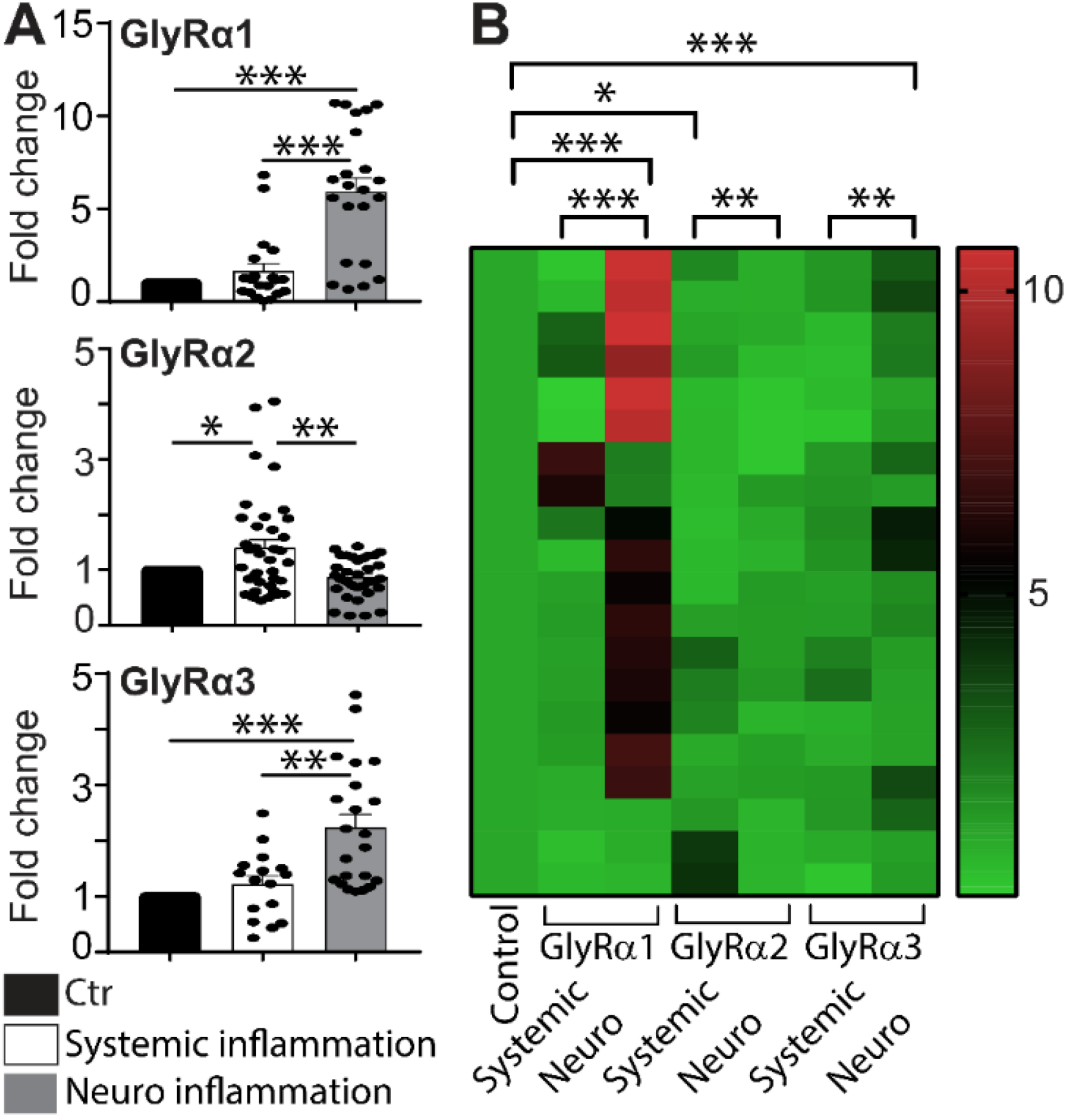
Neuronal GlyR response to systemic and neuroinflammation in mice. **(A)** Fold change in mRNA expression of GlyRα1, α2, and α3 in mouse brains (n=4-6 mice per group). Neuroinflammation induced upregulation of GlyRα1 and α3, with no change in GlyRα2 expression. Data are shown as mean ± SEM, calculated using the 2^^-ΔΔCt^ method. The experiment was performed twice in duplicate. Statistical significance was determined using one-way ANOVA, with *p<0.05, **p<0.01, and ***p<0.001. **(B)** Heat map displaying relative fold-change expression of GlyRα subunits in control, systemic, and neuroinflammatory brain samples. Control values were normalized to 1, with fold changes represented by the color scale (green = baseline/control, red = upregulation). Clustering showed marked upregulation of GlyRα1 and α3 during neuroinflammation.

GlyRα2 is a well-studied GlyR, especially in the brain. To determine whether brain GlyRα1, α2, and α3 respond differently to systemic and neuronal inflammation, mice were administered LPS intraperitoneally and intracortically (neuroinflammation), and samples were obtained 48 hours post-treatment. A group of untreated mice was enrolled as healthy controls. A significant increase in GlyRα3 (2.23 ± 0.2-fold, p<0.001**) expression in neuroinflammatory mice, along with the highest expression of GlyRα1 (5.91 ± 0.73-fold, p<0.001**) in neuro-inflammation compared to systemic inflammation and control was observed in brain tissues (**Figure 4A**).GlyRα2 expression was increased in response to systemic inflammation (1.4 ± 0.14, p=0.04*), with no change in neuroinflammation (**Figure 4A**). A heat map revealed up-regulation of brain GlyRα1 and α3 in response to neuroinflammation compared to systemic inflammation (**Figure 4B**). In contrast, GlyRα2 responds more to systemic inflammation than to neuroinflammation. The cytokines IL-1β, IL-2, and TNFα expression was also studied under inflammation conditions in the brain (**Supplementary Figure 2A**).

### Modulations of non-neuronal GlyR by systemic and neuronal inflammation

**Figure 5:**
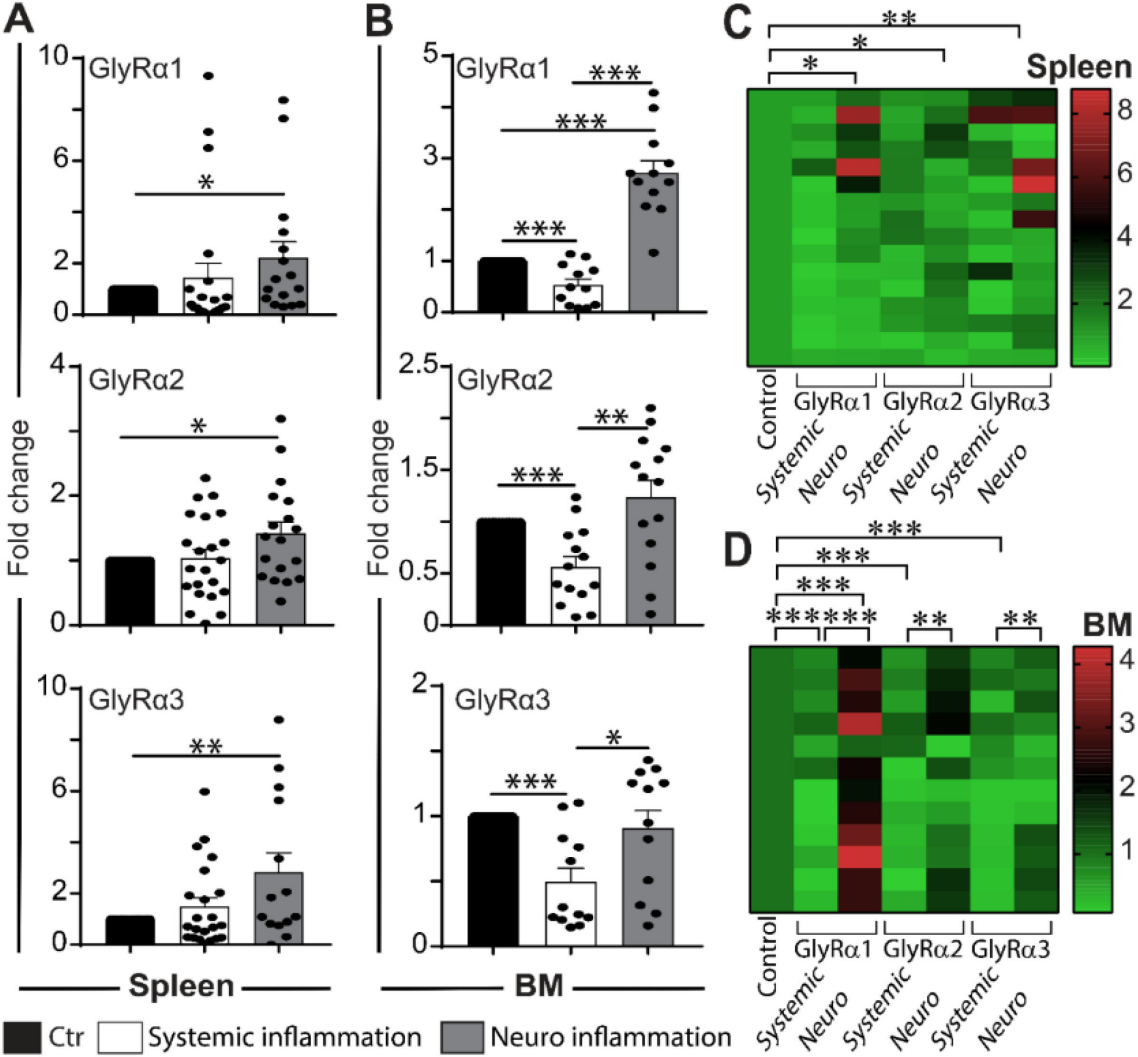
Non-neuronal (spleen and BM cells) GlyRα subunit expression during systemic and neuroinflammation. **(A)** Spleen tissue (n=4-6 mice per group) showing significant upregulation in GlyRα1, α2, and α3 under neuroinflammatory conditions, while remaining unchanged during systemic inflammation. This suggests enhanced splenic immune activation in response to central neuroinflammation. **(B)** BM cells (n=4-6 mie per group) displayed increased GlyRα1 and α2 expression during neuroinflammation, whereas systemic inflammation reduced the expression of all three GlyRα subunits. Data are presented as mean ± SEM using the 2^^-ΔΔCt^ method. Experiments were performed twice in duplicate. Statistical significance was determined by one-way ANOVA followed by Tukey’s *post hoc* test (*p<0.05, **p<0.01, and ***p<0.001). **(C, D)** Heat maps illustrated the relative fold-change expression profiles of GlyRα1, α2, and α3 in the spleen **(C)** and BM cells **(D)**. Control samples were assigned a baseline value of 1, with fold-change values (green = baseline/control, red = upregulation). The heat maps reveal distinct upregulation patterns of GlyRα subunits in both tissues, with stronger expression observed during neuroinflammatory conditions.

The spleen and BM from mouse models of systemic and neuronal inflammation were used to identify changes in the expression of GlyRα1, α2, and α3. Notably, neuroinflammation significantly increased GlyRα1 (2.22 ± 0.62, p=0.026*), GlyRα2 (1.41 ± 0.18, p=0.014*), and GlyRα3 (2.83 ± 0.76, p=0.004**) expression in spleen (**Figure 5A**) compared to the control. Systemic inflammation showed no significant changes in the spleen (**Figure 5A**). The association between systemic or neuronal inflammation and the changes in GlyR expression is represented as a heat map for the spleen (**Figure 5C**). During both the inflammations, spleen tissue showed increased IL-1β, IL-2, and TNFα expression (**Supplementary Figure 2B**). A significant splenic GlyR modulation was detected in neuroinflammation mice.

During neuroinflammation, the BM showed a significant increase in the expression of GlyRα1, α2, and α3 (**Figure 5B**, p<0.0001, p<0.01, p<0.05). In contrast, systemic inflammation reduced the expression of all three GlyRα subunits in the BM (**Figure 5B**). The BM showed reduced pro-inflammatory cytokine expression in systemic and neuroinflammation (**Supplementary Figure 5C**). The heat map represented the association of RNA overexpression in GlyRα1, α2, and α3 specifically in response to neuroinflammation (**Figure 5D**). Systemic inflammation-induced decreases of GlyR expression indicated a suppressive regulatory mechanism in the BM.

### Regulating circulating WBC GlyR by neuroinflammation

**Figure 6:**
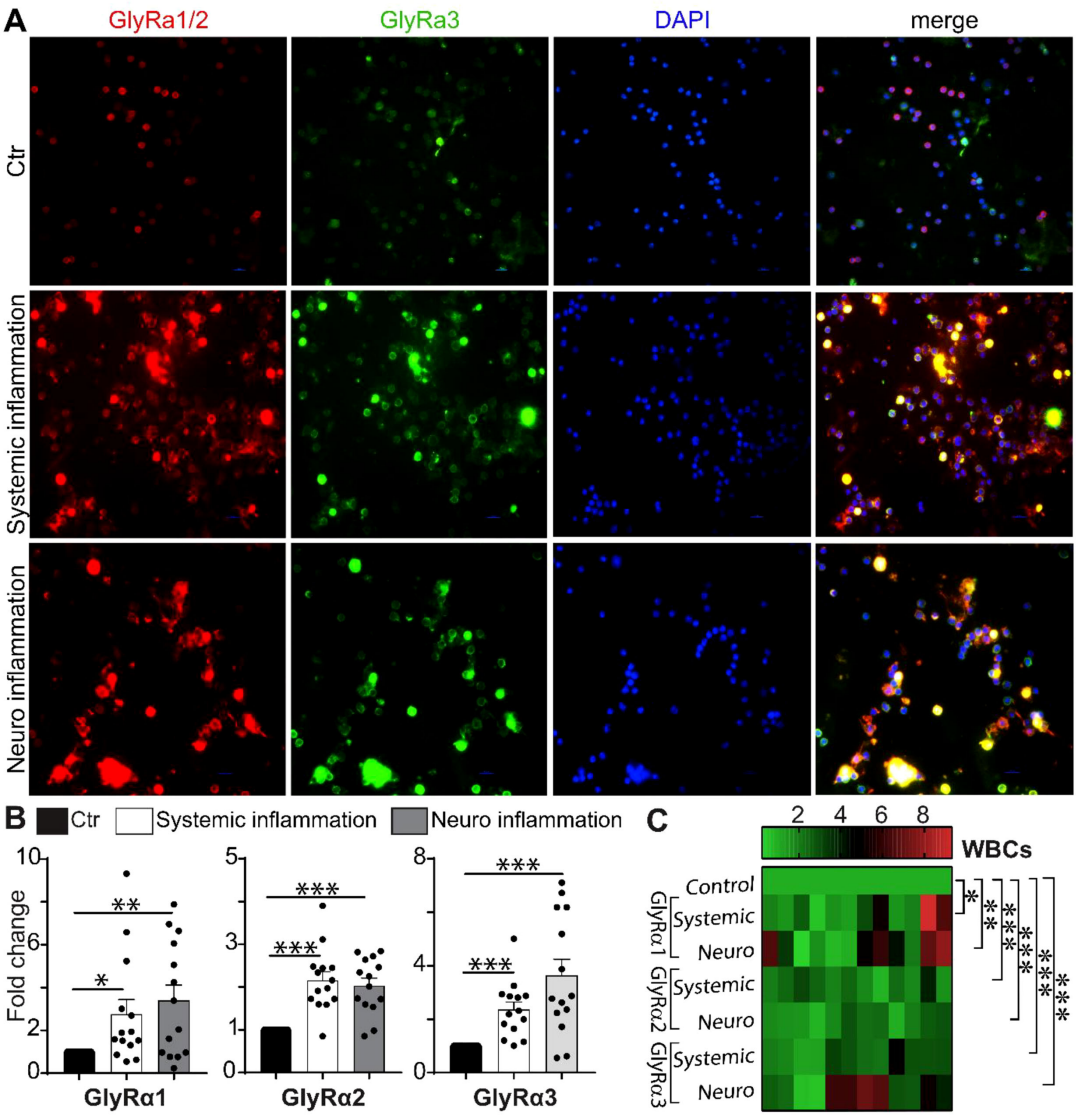
Blood-based GlyR response under different inflammatory conditions. **(A)** Representative immunofluorescence images showing GlyRα1/2 (red), GlyRα3 (green), and DAPI (blue) staining in WBCs from control, systemic- and neuroinflammation groups. *Scale bar: 10µm***(B)**. Fold-change expression of GlyRα1, α2, and α3 in circulating WBCs (n=4-6 mice per group) showed that neuroinflammation significantly upregulated all three GlyRα subunits, while systemic inflammation selectively increased GlyRα1 and α2 expression. This suggests enhanced peripheral glycinergic activation in response to neuroinflammation. The experiment was repeated twice in duplicates. Statistical significance was assessed by one-way ANOVA followed by Tukey’s *post hoc* test (*p<0.05, **p<0.01, and ***p<0.001). **(C)** Heat-map representing the fold-change expression profiles of GlyRα subunits in WBCs across different inflammatory conditions. The control group was normalized to 1, presented as green and red for upregulation. Distinct clustering patterns highlighted the strong upregulation of GlyRα subunits under neuroinflammation.

Immunofluorescence staining showed that inflammatory conditions influence the expression of GlyR subunits in circulating WBCs. In control WBCs, GlyRα1/2 and α3 signals were mainly expressed in the membrane. In contrast, a significant increase in GlyRα1/2 and α3 staining was observed in the cytoplasm and membrane in neuronal and systemic inflammation models (**Figure 6A**). The increased co-expression of GlyRα1/2 and α3 was observed in WBCs under inflammatory conditions.

Next, WBCs were collected from mouse models of neuronal and systemic inflammation. The naïve mice served as the controls. Both systemic and neuronal inflammatory conditions altered the expression of GlyRα1, α2, and α3 in WBCs (**Figure 6B**). The gene modification of circulatory WBC GlyR by neuroinflammation showed a significant increase in GlyRα1 (3.4 ± 0.72-fold, p=0.002**), GlyRα2 (2.03 ± 0.17-fold, p<0.0001**), and GlyRα3 (3.44 ± 0.61-fold, p=0.0003**) compared to the control (**Figure 6B**). GlyRα3 in WBCs revealed a higher correlation to neuroinflammation. The responses of GlyR to systemic and neuronal inflammation were compared to the control (**Figure 6C**). During both inflammations, WBCs showed decreased expression of IL-1β, IL-2, and TNF-α (**Supplementary Figure 2D**).

### Cytokine-independent regulation of WBC GlyR by neuroinflammation

**Figure 7:**
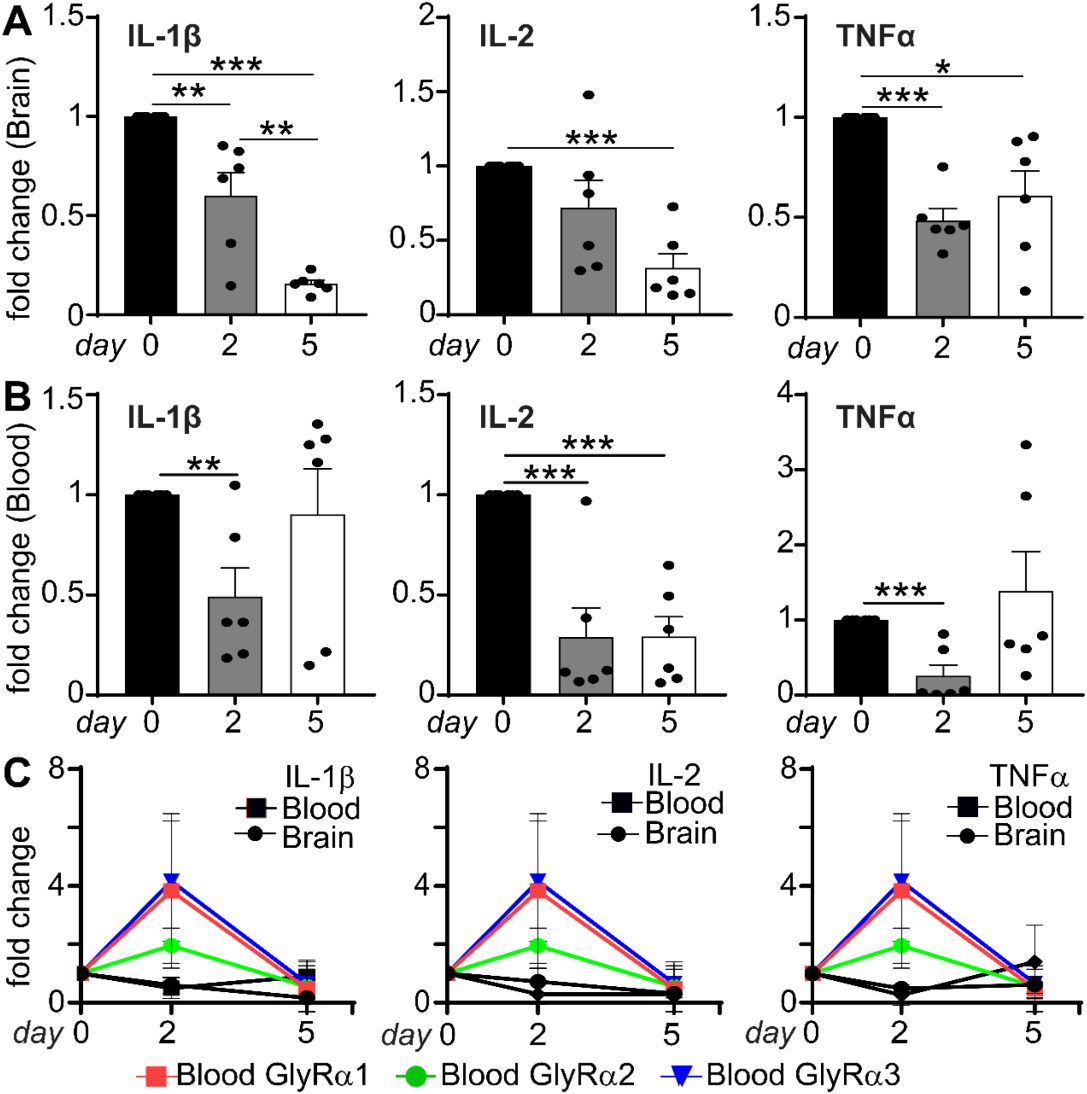
Cytokine expression dynamics during neuroinflammation in WBCs. **(A, B)** Fold-change in mRNA expression of proinflammatory cytokines, including IL-1β, IL-2, and TNFα, at days 0, 2, and 5 post-LPS treatment in the mouse brain and blood. A significant reduction in cytokine transcript levels was observed at day 2, further declining by day 5 compared with day 0 controls in the brain after neuroinflammation. **(C)** Line graphs illustrate the comparison of cytokine expression patterns in the brain and blood with blood GlyRα subunits, showing similar yet opposing trends, where the peak expression of peripheral GlyRα subunits on day 2 coincides with transient cytokine suppression in both tissues. This suggests reciprocal regulation between peripheral and central systems. Data are shown as fold change (mean ± SEM), calculated using the 2^^-ΔΔCt^ method. Statistical significance was determined by one-way ANOVA followed by Tukey’s *post hoc* test (*p<0.05, **p<0.01, and ***p<0.001).

The neuropathological process of neuroinflammation at days 2 and 5 altered the expression of GlyRs and pro-inflammatory cytokines in the opposite direction. A significant reduction in IL-1β, IL-2, and TNFα cytokines was observed in the brain (**Figure 7A**) and blood (**Figure 7B**) compared with the naive controls (day 0) following neuroinflammation at days 2 and 5. Pathological changes of neuroinflammation coincided with transient cytokine suppression in both the brain and blood. In contrast, a significant increase in WBC GlyRα1, a2, and a3 was observed on day 2, while a reduction of all three subunits of GlyR was seen following day 5. These results indicated that the increase in WBC GlyRs was not correlated with IL-1β, IL-1, and TNFα signaling in the brain and WBCs (**Figure 7C**). This demonstrated a reciprocal modulation of WBC GlyRs by neuroinflammation that is independent of brain and WBC cytokine signaling.

### Biomarker roles of WBC GlyRs in correlation with activated microglia

**Figure 8:**
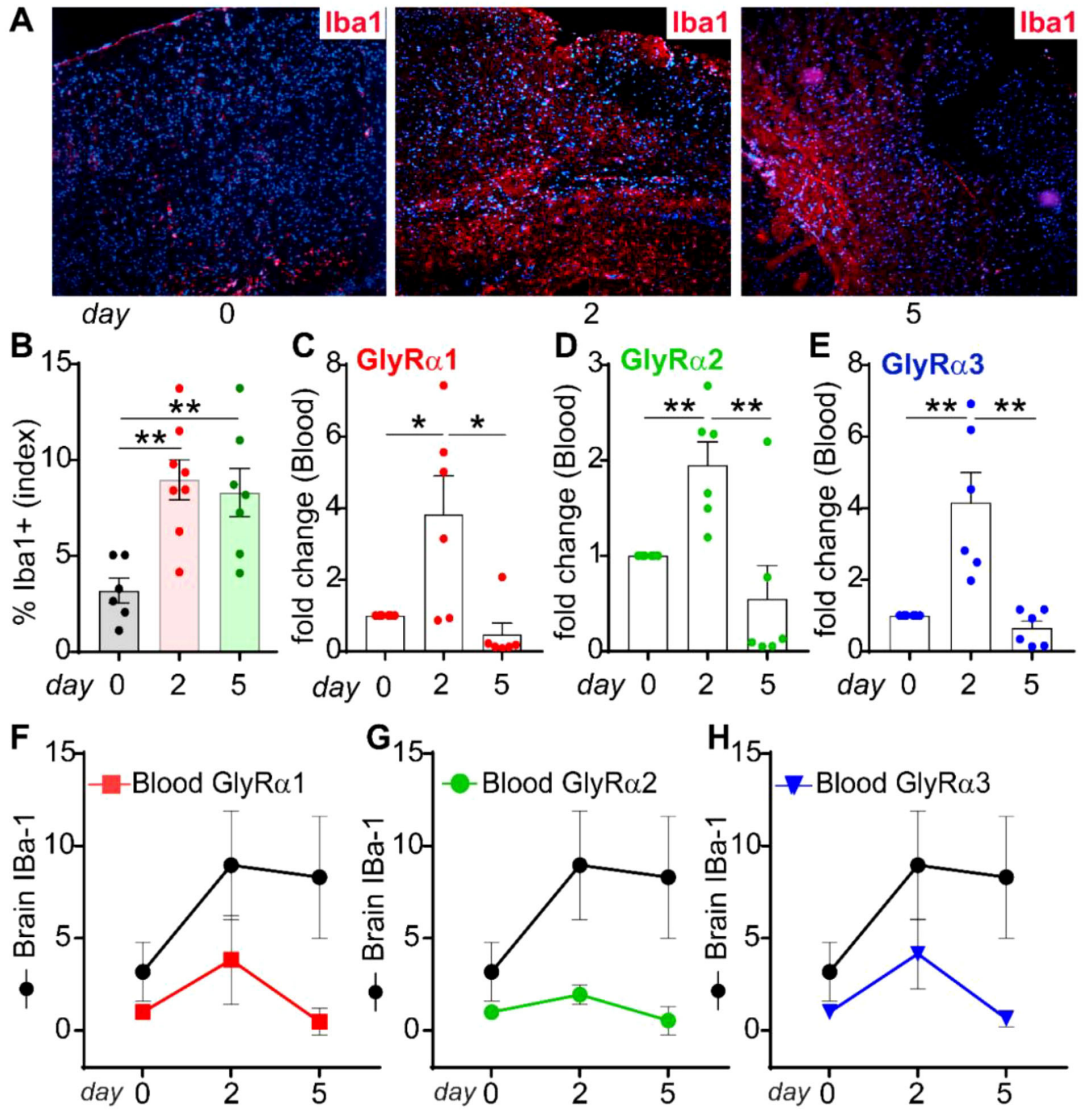
Blood-based biomarker function of GlyRs during neuroinflammation. **(A)** Representative immunofluorescence images of the left hemisphere cerebral cortex showing Iba1+ microglia (red) at day 0 (control), day 2, and day 5 following intracerebral neuroinflammation. Nuclei were counterstained with DAPI (blue). Iba1 immunoreactivity is markedly increased on day 2, indicating robust microglial activation. By day 5, Iba1+ microglial density partially decreases, suggesting resolution of the acute inflammatory response. Scale bar: 100 µm. **(B)** Quantification of Iba1+ microglial density (percentage index) in cortical sections confirms peak microglial activation at day 2, corresponding to the acute phase of neuroinflammation, with partial resolution by day 5. **(C–E)** GlyRα1, α2, and α3 expression in blood shows significant upregulation at day 2, followed by a decline to or below baseline levels by day 5. **(F–H)** Analysis of brain Iba1 (black lines) and blood GlyRα subunit (colored lines) expression during neuroinflammation reveals a coordinated central-peripheral association. **(F)** Iba1 and GlyRα1 elevation on day 2 reflects synchronized central-peripheral immune activation. By day 5, brain Iba1 remains moderately elevated while blood GlyRα1 declines below baseline, indicating rapid systemic resolution. **(G, H)** GlyRα2 and α3 exhibit similar transient upregulation at day 2, inversely correlating with the partial resolution of brain Iba1 by day 5, demonstrating a temporary reciprocal modulation between cortical microglia and peripheral GlyRα subunits. Data are presented as fold change (mean ± SEM), calculated using the 2^^-ΔΔCt^ method. Experiments were performed twice in duplicate. Statistical significance was determined by one-way ANOVA followed by Tukey’s *post hoc* test (*p<0.05, **p<0.01, and ***p<0.001).

Next, we investigated how neuropathological changes affected the WBC GlyRs under neuroinflammatory conditions. Pathological evaluation was performed using an immunofluorescence assay in the brain slides with an antibody against Iba1. The naïve brain sections were used as the controls (day 0). Immunofluorescence imaging of the LPS-injected left hemisphere cerebral cortex showed a significantly increased activation of Iba1+ microglia (**Figure 8A**, red) at days 2 and 5 of post-LPS injection compared to the controls. The neuropathological change in Iba1+ microglia reflects neuroinflammation induced by LPS, with DAPI-counterstained nuclei (**Figure 8A**, blue). A noticeable increase in Iba1+ microglial density evidenced the neuropathological process caused by LPS on days 2 and 5. Quantification of Iba1+ cell density (percentage index) in brain sections indicated that robust acute microglial activation occurred on day 2 and persisted through to day 5 (**Figure 8B**).

The mRNA expression of GlyRα1 (3.82 ± 1.08-fold, p=0.025*), GlyRα2 (1.95 ± 0.24-fold, p=0.022**), and GlyRα3 (4.15 ± 0.84-fold, p=0.003**) in WBCs showed a statistically significant increase at day 2, following a decreased WBC GlyRs at day 5 (**Figures 8C–E**). This coincided with elevated microglial activation. The early peak in GlyR expression in WBCs reflects a dynamic peripheral response mediated by brain microglia activation. These results provide evidence that WBC GlyR signaling occurs in response to neuropathological gliosis through unexplored or glycinergic signaling mechanisms.

Finally, we analyzed whether circulating WBC GlyR signaling correlated with activation of Iba1+ microglia to identify the brain-GlyR-WBCs communication axis. The communication was reflected by pathological changes in Iba1+ microglia (neuroinflammation) and the expression of GlyRα1, α2, and α3 in circulating WBCs (**Figures 8F–H**). With a transient rise in activation of Iba1+ microglial cells, increases in GlyR expression tend to be high on day 2, suggesting a crosstalk of brain-GlyR-blood during neuroinflammation. Brain Iba1 and blood GlyRα1 and α3 show an acute surge on day 2, indicating synchronized central-peripheral immune activation. While brain Iba1 remains moderately elevated at day 5, blood GlyRα1 and α3 expression sharply decline, suggesting rapid systemic normalization following peak inflammation (**Figures 8F, H**). GlyRα2 transcript exhibits a slight early upregulation on day 2, inversely correlating with the decline in brain Iba1 expression by day 5 (**Figure 8G**). These findings demonstrate that LPS-induced neuroinflammation leads to rapid microglia activation accompanied by a synchronized, transient upregulation of circulating WBC GlyRα1 and α3. The reciprocal temporal association suggests that circulating WBC GlyR expression may serve as a potential biomarker for neuroinflammation.

## Discussion

Neuroinflammation is a serious condition in the central nervous system, triggered by various factors, including infectious diseases, traumatic injuries, or neurodegenerative disorders (25). Depending on the cause, inflammation can range from acute (caused by pathogens or traumatic brain injury) to chronic, affecting disease progression and patient outcomes. Neuroinflammation involves complex interactions among neurons, glial cells, and infiltrating immune cells (26, 27). These neuronal cells are essential not only for neurotransmission but also for maintaining CNS balance and defending against pathogens and damage. Neuroinflammation-mediated activation of microglia and astrocytes leads to the release of inflammatory cytokines, which can have both protective and harmful effects (28). Currently, diagnosing neuroinflammation primarily relies on expensive neuroimaging or invasive cerebrospinal fluid (CSF) analysis. Common biomarkers, including glial fibrillary acidic protein (GFAP), chitinase-3-like protein 1 (YKL-40), soluble triggering receptor expressed on myeloid cells 2 (sTREM2), and neurofilament light chain (NfL) (20, 23), mainly reflect glial activation or axonal injury rather than dynamic immune-neuronal communication. These limitations underscore the need for minimally invasive biomarkers that can assess active neuroimmune interactions, thereby enhancing early diagnosis and therapeutic monitoring.

Glycine receptors, traditionally known as inhibitory neurotransmitter receptors in the CNS, are increasingly recognized as key regulators of inflammation. GlyRs are essential for neurophysiological functions such as motor coordination, respiratory rhythm, and muscle tone, and they have been associated with various pathological conditions (29). Our data confirmed the presence of GlyRα1, α2, and α3 transcripts in both PBMCs and monocytes and, for the first time, showed that GlyRα1 was the dominant subtype in these immune cells. These results are consistent with Eynden et al. (24), who reported GlyRα2 expression in a subset of PBMCs, with predominant expression in CD14+ monocytes, and substantial expression in CD56+ natural killer (NK) cells. This indicates that peripheral immune cells may have a broader range of GlyR subunits than previously thought.

In the present study, immunofluorescence analysis showed dual membrane-associated and cytoplasmic localization of GlyRα1/2 subunits in CD14+ monocytes. Such dual localization is well-documented in neurons, where surface localization of GlyR subunits is tightly regulated, but this process is not well understood in immune cells (30). Possibly, membrane-bound GlyRs enable rapid responses, while cytoplasmic GlyRs might act as intracellular reservoirs, potentially performing functions similar to those in neuronal cells of the CNS. Similar cytoplasmic location has been described in oligodendroglial cell lines (MO3.13, OLN-93, HOG) and spinal cord neurons (31). GlyRα1 is widely expressed in the spinal cord and brainstem, and genetic changes in GlyRα1 and β-subunits are linked to human hyperekplexia, leading to lower pain thresholds (32). GlyRα1 and GlyRα3 are mainly neuronal, while GlyRα2 is broadly expressed during development and in non-neuronal tissues (9). Single-cell transcriptomic analyses demonstrate low but widespread GlyRα3 expression across excitatory and inhibitory neurons in the mouse CNS, with marked regional and sex-specific differences (21). Microglial cells were found to harbor functional GlyRα1β when studied ex vivo in spinal cord slices (33, 34). However, *in vitro* studies reported GlyR downregulation, possibly due to excess glycine in cell culture medium, which is linked to cell death in heterologous GlyR expression experiments (35). This differential expression suggests that extraneuronal GlyRs may play a role in immune system regulation, contributing to glycinergic control in both excitatory and inhibitory processes.

Beyond the CNS, glycine-mediated cytoprotective and immunomodulatory effects have been documented in several non-neuronal tissues, including the kidney, liver, heart, and endothelial cells (32). Pan C et al. (36) proposed that GlyRα1 mediates protection against ATP-depletion-induced injury in human embryonic kidney (HEK293) and Madin-Darby canine kidney (MDCK) cells. A GlyRβ subunit splice variant has been detected in liver homogenates, with unknown cellular origin (37), whereas cardiomyocytes have been shown to express GlyRβ, but not GlyRα subunits, in an experimental model of LPS-induced cardiac dysfunction (hypoxia/reoxygenation injury) (38). Glycine supplementation (2 mM) confers cardioprotection, highlighting a functional role for non-neuronal GlyRs. These non-neuronal cells expressing GlyRs mediate glycine’s cytoprotective and modulatory effects, with glycine acting as the inhibitory neurotransmitter of GlyRs.

The significance of GlyRs in immune activation was demonstrated through experiments involving LPS stimulation in human monocytes, mouse BMMs, and mouse inflammation models. *In vitro* studies showed early upregulation of GlyRα1 during acute inflammation and a delayed increase in GlyRα3 during the anti-inflammatory phase. This time-dependent expression of GlyRα1 and GlyRα3 induced by LPS, along with peak levels of pro-inflammatory cytokines, suggests that GlyRs might serve as inhibitory feedback regulators, modulating immune responses to prevent excessive cytokine release. Similarly, Mulvey et al. (39) described an early pro-inflammatory response followed by anti-inflammatory processes in LPS-treated THP-1 cells. The concurrent high expression of GlyRα3 with anti-inflammatory cytokines points to a potential role in resolving inflammation. Glycine-mediated inhibition of TNF-α and IL-1β production in LPS-treated human monocytes supports these findings (40), linking peak GlyRα1 expression with increased IL-1β and TNF-α levels, indicating a compensatory role for GlyRα1 during acute immune activation. GlyRα2 showed the highest baseline expression in the brain, aligning with its role in inhibitory neurotransmission in cortical and subcortical circuits (21). In contrast, peripheral tissues displayed variable expression patterns, with GlyRα2 being predominant. Neuroinflammation selectively upregulated GlyRα1 and GlyRα3 in brain tissues, whereas systemic inflammation primarily increased GlyRα2 expression.

In the spleen, a major secondary lymphoid organ, a significant upregulation of GlyRα1 and GlyRα3 mRNA may reflect heightened activation of splenic macrophages and monocytes during inflammation. This selective, differential upregulation implies that individual GlyRα subunits may have specific regulatory roles in modulating splenic immune responses. These findings align with previous reports indicating that splenic macrophages express functional GlyRs, which help suppress pro-inflammatory cytokine production and influence immune cell function (21, 41). Froh M. et al. (42) first demonstrated that splenic macrophages primarily express the GlyRα2 and Kupffer cells express GlyRα1 along with the common α4 and β subunits in adult mice. Li et al., 2001 (43) showed that glycine suppresses LPS-induced TNF-α production, NF-κβ activation, and oxidative burst in macrophages, thereby confirming the functional presence of GlyRs in splenic immune cells and establishing glycine as an anti-inflammatory immunomodulator.

Our results reveal a distinct pattern of GlyRα subunit regulation in BM cells, with an increase in GlyRα1 expression during neuroinflammation, contrasting with the suppression of all GlyRα subunits during systemic inflammation. This differential regulation may serve as a feedback mechanism to maintain immune homeostasis. Although direct characterization of GlyRs in BM-derived cells remains limited, emerging evidence supports glycinergic signaling as a broader immunoregulatory mechanism that influences immune progenitor differentiation and macrophage polarization (17). Conversely, the downregulation of all GlyR subunits during systemic inflammation may indicate exhaustion or suppression of glycinergic anti-inflammatory tone under sustained peripheral immune activation. Neuroinflammation also prompted simultaneous upregulation of GlyRα subunits in the spleen, bone marrow, and circulating WBCs, along with suppression of CNS cytokines. This reciprocal pattern suggests a potential brain-to-blood signaling axis, in which peripheral GlyRs may serve as early responders to CNS inflammatory states (27, 44).

LPS-based neuroinflammation model enables identification of non-neuronal GlyR signaling mechanisms, specifically the neuropathogenic modification of GlyR signaling in peripheral immune tissues, especially in circulating WBCs. In immune cells such as macrophages, particularly Kupffer cells, GlyRα1, α4, and β subunit transcripts and proteins were detected (42). Rat neutrophils express functional GlyRα2, α4, and β subunits, which inhibit LPS-induced calcium fluxes by glycine (42). Human monocytes and NK cells express GlyRα2, which inhibits LPS-induced (endotoxin shock) TNFα and IL-1β secretion, and increases IL-10 production by glycine (40). Furthermore, subunit-specific expression patterns reported in macrophages from different tissues suggest potential heterogeneity in GlyR composition across immune cell populations, which could underlie distinct signaling properties.

The present study demonstrates that neuroinflammation (IC administration of LPS) induces a robust but transient microglia activation (increased Iba1 immunoreactivity) in the cerebral cortex that peaks at day 2 and gradually resolves by day 5. This localized neuroinflammatory response was accompanied by a parallel, transient upregulation of peripheral blood GlyRα1–3 subunit mRNA expression. This temporal pattern closely resembles the time course of cortical microglial activation, suggesting dynamic-to-central-peripheral immunological crosstalk and identifying GlyRα subunits as potential peripheral indicators of central neuroinflammatory activity.

Microglial activation after focal LPS injection or local injury (45) induces an early proinflammatory phase dominated by cytokine production that transitions into a recovery phase. The intracortical route limits LPS diffusion, thereby limiting strong peripheral cytokine induction (46). The positive correlation between WBC GlyR expression and brain Iba1+ activation at day 2 reflects systemic immune responses, as GlyR-expressed peripheral immune cells mirror neuroinflammatory events. Another possible mechanistic link between brain and blood GlyR regulation may involve exosomal communication (47), where microglia and astrocytes release extracellular vesicles containing RNA and protein that can enter circulation and modulate gene expression in peripheral cells, linking central inflammation to peripheral changes.

Several limitations lead to the need for further study of non-neuronal GlyR signaling. First, while we demonstrated transcriptional and protein expression of GlyRs under normal and inflammatory conditions, the validation cohorts were mainly limited to human tissue samples. Lack of GlyRα subunit-specific modification of models restricts the mechanistic evaluation of GlyR signaling pathways, especially in human neuroinflammatory diseases, including virus-induced encephalitis and multiple sclerosis. Need for a full cytokine panel assessment to strengthen conclusions regarding cytokine-independent regulation of circulating immune cell GlyRs during neuroinflammation. Nevertheless, the potential sex-specific differences in GlyR expression cannot be excluded. The mechanism connecting central neuroinflammation to peripheral GlyRs upregulation remains unclear; therefore, it warrants further investigation.

## Conclusion

Neuroinflammation contributes to diverse brain disorders, but minimally invasive biomarkers for its detection are still limited. We discovered that GlyRs, best known for their inhibitory function in the CNS, are constitutively expressed in circulating WBCs, with their expression dynamically modulated during neuroinflammation. Our findings demonstrate a brain-blood GlyR axis, linking central neuronal signaling with peripheral immune responses, suggesting GlyRs as potential blood-based biomarkers for the early detection and monitoring of neuronal inflammatory states.

## Data Availability

The original contributions presented in the study are included in the article/**Supplementary Material**. Further inquiries can be directed to the corresponding author.
