# Supplementary material for: Glycine receptors in circulating white blood cells regulated by neuroinflammation
Source: Front Immunol. 2026 Feb 23;17:1749275. doi: 10.3389/fimmu.2026.1749275 (PMC12967963; doi:10.3389/fimmu.2026.1749275)
Supplement: Supplementary file 1 [file DataSheet1.docx]

**Supplementary Figure and legend**


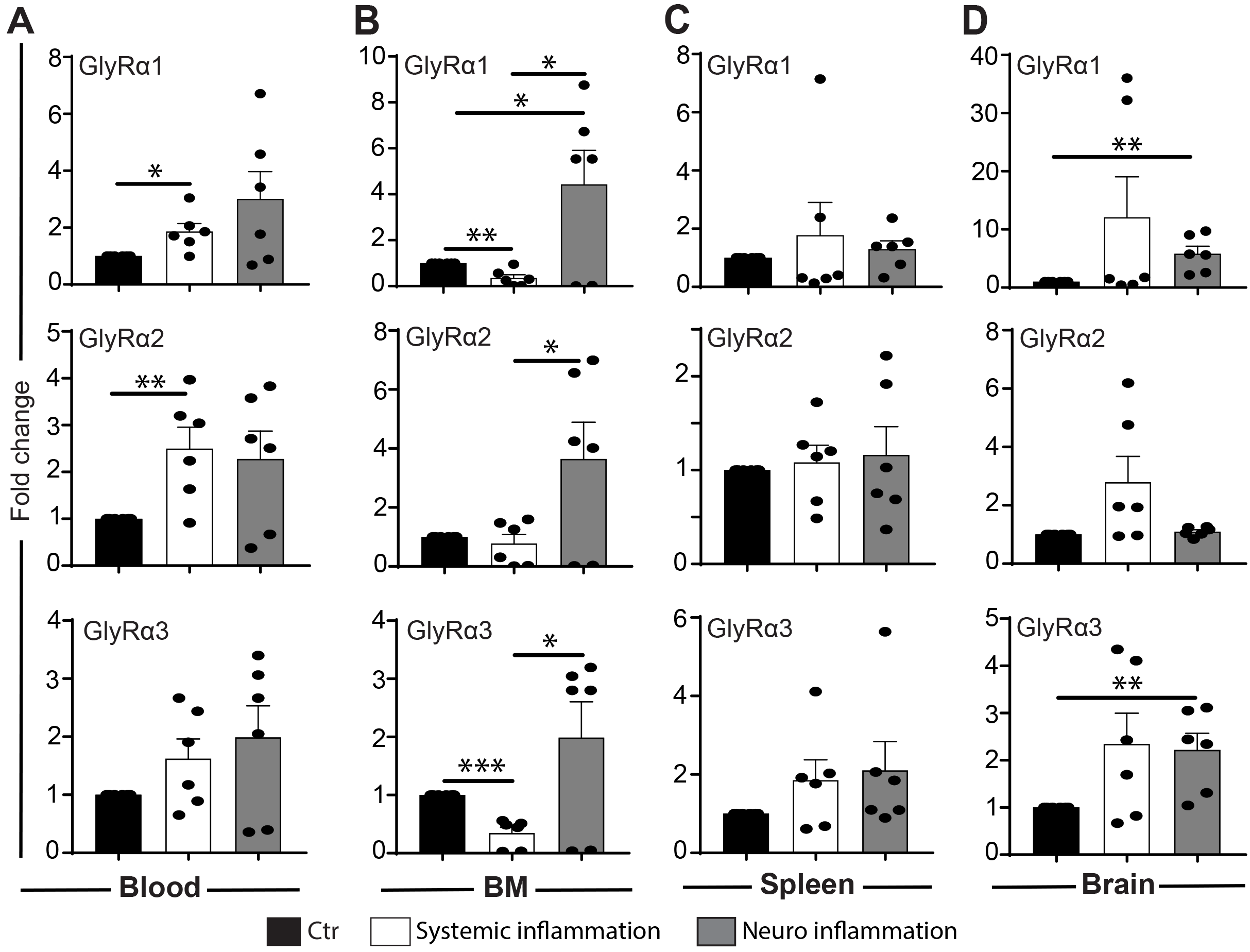


**Supplementary Figure 1:** GlyRs subunit expression during systemic and neuroinflammation in male mice in blood (A), BM (B), Spleen (C), and Brain (D). Data are presented as mean ± SEM (n=3 per group), calculated using the 2^^-∆∆Ct^ method. The experiment was performed in duplicate. Statistical significance was performed using one-way ANOVA followed by Tukey’s post hoc test (*p<0.05, **p<0.01, and ***p<0.001).

**eg**


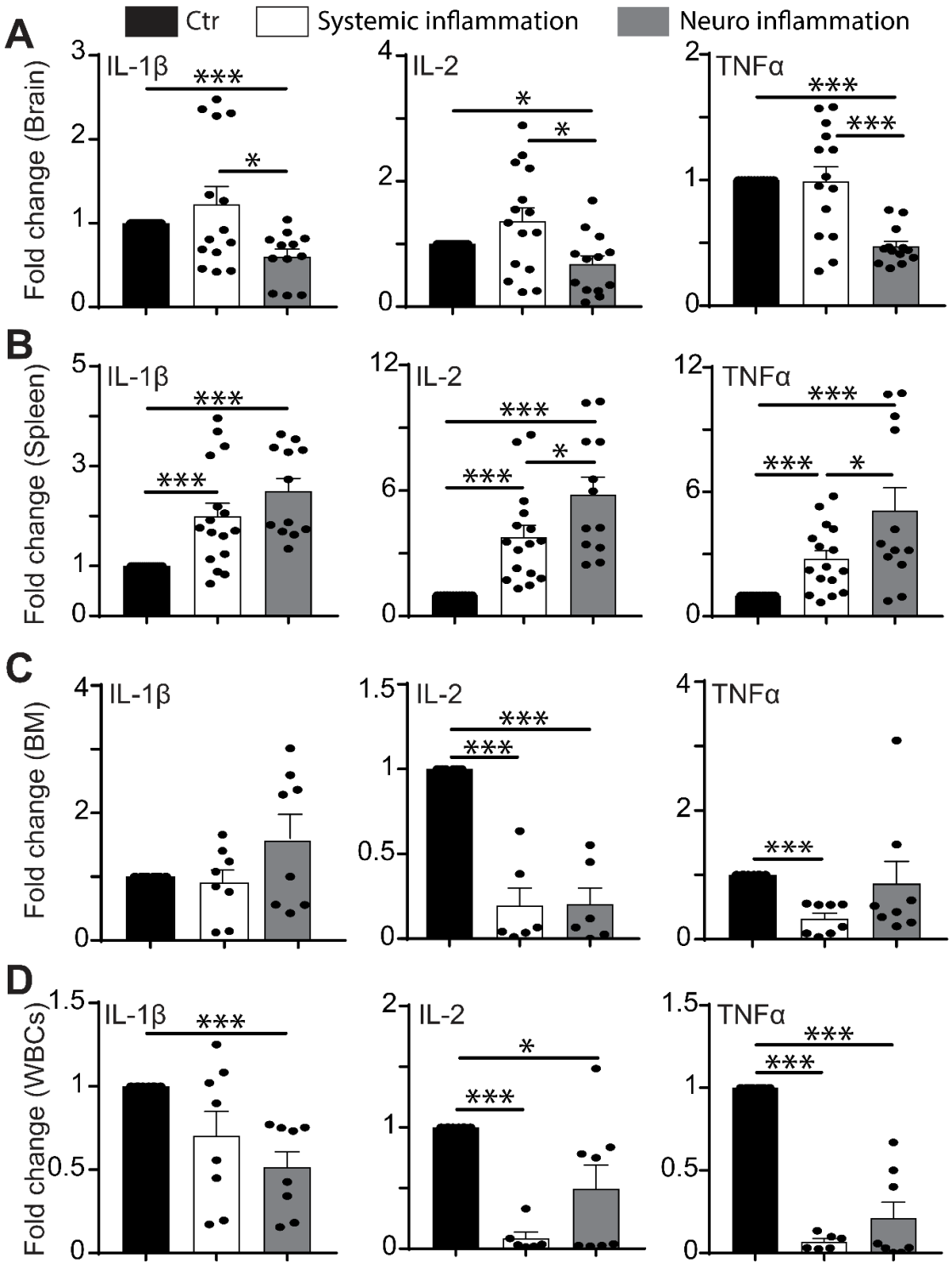


**Supplementary Figure 2:** Tissue-specific cytokine responses during systemic and neuroinflammation in mice. **(A)** Brain IL-1β, IL-2, and TNFα expression decreased during neuroinflammation compared to systemic inflammation and control. (**B**) Spleen showed significant upregulation of cytokines under both inflammatory conditions, suggesting enhanced splenic immune activation. **(C-D)** BM and WBCs showed a reduction in cytokine transcripts during systemic and neuroinflammation, indicating GlyRα-mediated immunosuppression. Data represented as mean ± SEM (n=3-4 per group), calculated using the 2^^-∆∆Ct^ method. The experiment was performed twice in duplicate. Statistical significance was performed using one-way ANOVA followed by Tukey’s post hoc test (*p<0.05, **p<0.01, and ***p<0.001).
